# A Role for GDNF and Soluble APP as Biomarkers of Amyotrophic Lateral Sclerosis Pathophysiology

**DOI:** 10.3389/fneur.2018.00384

**Published:** 2018-05-30

**Authors:** Serena Stanga, Liliana Brambilla, Bernadette Tasiaux, Anh H. Dang, Adrian Ivanoiu, Jean-Noël Octave, Daniela Rossi, Vincent van Pesch, Pascal Kienlen-Campard

**Affiliations:** ^1^Alzheimer Research Group, Institute of Neuroscience, Université Catholique de Louvain, Brussels, Belgium; ^2^Laboratory for Research on Neurodegenerative Disorders, Istituti Clinici Scientifici Maugeri SpA SB - IRCCS, Pavia, Italy; ^3^Unité de Neurochimie, Institute of Neuroscience, Université Catholique de Louvain, Brussels, Belgium; ^4^Neurology Department, Cliniques Universitaires Saint-Luc, Brussels, Belgium

**Keywords:** biomarker, neurodegeneration, glial cell line-derived neurotrophic factor (GDNF), amyloid precursor protein (APP), amyotrophic lateral sclerosis (ALS)

## Abstract

The current inability of clinical criteria to accurately identify the “at-risk group” for Amyotrophic Lateral Sclerosis (ALS) development as well as its unknown etiology are fueling the interest in biomarkers aimed at completing clinical approaches for the diagnosis. The Glial cell line-derived neurotrophic factor (GDNF) is a diffusible peptide critically involved in neuronal differentiation and survival. GDNF is largely studied in various neurological and neuromuscular diseases, with a great interest in the peripheral nervous system (PNS). The recent discovery of Amyloid Precursor Protein (APP)-dependent GDNF regulation driving neuro-muscular junctions' formation in APP null transgenic mice, prompts to study whether neurodegeneration relies on loss or gain of APP function and suggests that it could affect peripheral processes. Here, we explored a brand-new aspect of the loss of trophic support in ALS by measuring GDNF, APP, soluble APP fragments and Aβ peptides levels in SOD1^WT^ or SOD1^G93A^ transgenic mouse models of ALS and in human biological fluids [i.e. serum and cerebrospinal fluid (CSF)] from ALS patients and control subjects. Our results show that both GDNF and soluble APP fragments levels are altered at the onset of motor deficits in mice and that their levels are also modified in patient samples. This study indicates that both GDNF and soluble APPα represent possible biomarkers for ALS.

## Introduction

Amyotrophic lateral sclerosis (ALS) is a neurodegenerative disease characterized by the selective and progressive loss of motor neurons from the motor cortex, brain stem, and spinal cord leading to muscle atrophy, irreversible paralysis and eventually death within 3–5 years from symptom onset as a result of respiratory failure. Most patients are aged between 50 and 75 years at diagnosis and ALS is mostly sporadic with 90% of the cases occurring without a family history of the disease. Although the disease is considered a rare type of motor neuron neurodegeneration, by 2040 it has been estimated that around 400,000 patients will be diagnosed with ALS worldwide (1). Currently approved treatments for the disease, i.e. Riluzole and Edaravone, extend survival by few months and only mildly improve motor function. The inefficacy of the available treatments may be attributed to the fact that ALS is a heterogeneous disease. Furthermore, the process of diagnosis is often delayed, mainly because many parameters need to meet diagnostic criteria.

Understanding the pathophysiology of both familial and sporadic ALS and finding specific biomarkers to accelerate diagnosis could help in developing more effective treatments. Trophic factors have been largely studied as potential therapeutic targets for ALS because of their essential role in neuronal development, motor neuron maintenance and survival (2). Difficulties for growth factor-based therapies relate to the fact that therapeutic intervention mostly occurs after the diagnosis, thereby resulting as ineffective in counteracting the already ongoing neurodegenerative process. In this regard, trophic factors' levels would rather be useful as biomarkers for diagnosis and/or prognosis.

Interestingly, aside from their synthesis in the local spinal/muscular microenvironment, trophic factors play important roles in the nourishing feedback during which originating neurons receive trophic input from their target tissues (3). In ALS, it has been proposed that the failure of muscle cells to release neurotrophic factors that maintain the favorable physiological context for spinal motor neurons may lead to the loss of that neuronal cell population (4).

The glial cell line-derived neurotrophic factor (GDNF) is one of the factors produced by muscles and Schwann cells. The absence of GDNF alters the location of developing spinal motor neurons that innervate the limbs (5) and selectively affects the innervation of intrafusal muscle spindles in mice (6). Interestingly, the overexpression of this factor in muscle during development causes a hyperinnervation of neuromuscular junctions (7), while GDNF heterozygous mice (+/−) exhibit locomotor deficiencies (8), suggesting that GDNF dosage plays a key role in neuromuscular function. When GDNF is administered directly in muscles, it preserves the muscle-nerve synapse and promotes motor neuron function and survival in a familial rat model of ALS (9). Furthermore, overexpression of GDNF in muscle extends lifespan in ALS mice (10). Interestingly, GDNF can be retrogradely transported along motor neuronal axons (11), thereby enabling to explore the relevance of the intramuscular delivery route to act on both somas and nerve endings.

Recently, we demonstrated that the Amyloid Precursor Protein (APP) controls GDNF transcription in mouse embryonic fibroblasts (MEFs) and muscles with an important impact on muscular trophy and on the formation of neuromuscular contacts (12). Together with others, our study suggests that defects in APP function might be directly related not only to Alzheimer's disease (AD), but also to other neurodegenerative diseases involving muscle denervation, such as ALS. Indeed, APP is upregulated in muscle from mouse models of familial ALS as well as from patients with ALS, where APP upregulation correlates with clinical symptoms (13, 14).

In this study, we investigated in parallel the expression levels of GDNF and APP and its metabolites in muscles from non-transgenic mice and transgenic mice overexpressing the wild-type human isoform of the Cu/Zn superoxide dismutase 1 (SOD1^WT^) enzyme or the mutant SOD1^G93A^ protein, the latter animals recapitulating much of the pathophysiology of ALS. We analyzed APP processing by measuring the levels of its soluble non-amyloidogenic fragments (sAPPα and sAPPβ) and amyloid-β (Aβ peptides), together with GDNF levels, in cerebrospinal fluid (CSF) and serum from ALS patients and healthy controls.

We found that both GDNF and APP levels are increased in hindlimbs muscles of the transgenic SOD1^G93A^ mouse model of ALS at the onset of motor deficits. Alterations in GDNF and sAPPα levels have been observed also in human biological fluids from patients with a moderate and fast progression of the disease. More precisely, GDNF levels are importantly decreased in the serum of ALS patients while sAPPα levels are increased in the same fluid, compared to healthy controls.

Altogether, our results suggest that both GDNF and sAPPα, which are clearly involved in neuromuscular pathologies, represent possible biomarkers for ALS pathophysiology.

## Materials and methods

### Mouse models and genotyping

Transgenic mice expressing human SOD1^WT^ [B6SJL-Tg(SOD1) 2Gur/J–002298] or SOD1^G93A^ [B6SJL-TgN(SOD1-G93A) 1Gur/J–002726] were purchased from The Jackson Laboratories. Animal care and handling were performed according to the European Council Directive 2010/63/EU and to the Italian and Belgian Animal Welfare Act for the use and care of laboratory animals and approved by the Animal Ethics Committee of the Universite Catholique of Louvain.

### Genotyping and tissue processing

Genomic DNA was extracted from mouse tail biopsies and used as DNA template in genotyping PCR analysis. Offspring positive for the SOD1^G93A^ transgene were identified using the following primers: SOD1, forward 5′-CATCAGCCCTAATCCATCTGA-3′ and reverse 5′-CGCGACTAACAATCAAAGTGA-3′ as described in Brambilla et al. (15). Hindlimbs muscles (gastrocnemius and tibialis anterior) from transgenic mice were snap frozen in liquid nitrogen and stored at −80°C until further use (RNA isolation for RT-qPCR and Western blotting).

### RNA preparation, RT-PCR, and quantitative PCR

RNAs were extracted from tissues in TRIzol Reagent and reverse transcribed using an iScript cDNA Synthesis Kit (Bio-Rad). Real-time quantitative PCR (qPCR) analysis was performed on 2ng cDNA template by using iQ SYBR Green Supermix in an iCycler IQ Multicolor Real-Time PCR Detection System (Bio-Rad). qPCR conditions were typically 95°C for 30 s, followed by 40 cycles of 30 s at 95°C, 45 s at 60°C, and 15 s at 79°C and ended by 71 cycles of 30 s at 60°C. The relative changes in the target gene: glyceraldehyde 3-phosphate dehydrogenase mRNA ratio were determined by the 2^(−ΔΔCt)^ calculation.

The sequences for qPCR primers are the following: GDNF, forward 5′-TTAATGTCCAACTGGGGGTCT-3′ and reverse 5′-GCCGAGGGAGTGGTCTTC-3′; and glyceraldehyde 3-phosphate dehydrogenase, forward 5′-ACCCAGAAGACTGTGGATGG-3′ and reverse 5′-ACACATTGGGGGTAGGAACA-3′.

### Western blotting

Muscles were homogenized in 10 volumes of lysis buffer [50 mM Tris (pH 7.5), 150 mM NaCl, 0.05 mM EDTA, 1% Triton X-100, and 0.1% sodium dodecyl sulfate] with Complete Protease Inhibitor Cocktail on ice using a Ultraturax homogenizer 3 × 10 s for each sample and in between 30 s (or more) on ice to cool down. After 1h at 4°C with continuous rotation, samples were centrifuged at 10,000 g for 10 min at 4°C and the supernatants were collected. Protein concentration was determined by the BCA Protein Assay Kit (Bio-Rad). A total of 15 μg protein was heated for 10 min at 70°C in loading buffer (lysis buffer containing 0.5 M DTT and staining NuPAGE blue), loaded and separated onto NuPAGE4–12% Bis-TrisGel, and then transferred for 2 h at 30 V onto PVDF membranes. After blocking (5% nonfat milk in PBS-Tween 0.05%), membranes were incubated overnight at 4°C with the primary antibodies, washed, and incubated with the secondary antibody coupled to peroxidase prior to ECL detection (GEHealthcare). ECL signals were quantified with a GelQuantNET software. Primary antibodies included anti-APP Y188 (1:5,000) or GAPDH_2_ (1:25,000) in 5% nonfat milk in PBS-Tween 0.05%. Secondary antibody included anti-rabbit (1:10,000).

### Study group and sampling

Venous serum samples and CSF were obtained from a group of subjects consisting of 7 patients with sporadic ALS and 7 healthy age-matched controls (CTR); clinical and demographic features are summarized in Table 1. All the subjects were examined by a senior neurologist and diagnosis of ALS was made according to the El Escorial criteria (16). ALS was diagnosed based upon symptom evaluation, neurological examination, laboratory and brain and/or spinal cord magnetic resonance imaging. Disease progression rates have been calculated as the ratio between the functional scale and disease duration in months; < 0.5 is considered slow; 0.5–1: moderate and >1: fast progression.

**Table 1:** Demographic and clinical variables of the study group.

**Patients**	**Age range at collection (years)**	**Onset**	**L.O.I. until diagnosis (months)**	**ALSFRS-R score**	**Progression rate (point/month)**
Control 1	56–61	NA	NA	NA	NA
Control 2	60–65	NA	NA	NA	NA
Control 3	40–45	NA	NA	NA	NA
Control 4	60–65	NA	NA	NA	NA
Control 5	70–75	NA	NA	NA	NA
Control 6	50–55	NA	NA	NA	NA
Control 7	50–55	NA	NA	NA	NA
ALS 1	56–61	Bulbar	1	44	44
ALS 2	36–41	Bulbar + hemiparesis	6	43	7.2
ALS 3	66–71	Paraparesis	48	38	0.8
ALS 4	60–65	Bulbar + hemiparesis	8	37	4.6
ALS 5	70–75	Paraparesis	11	43	3.9
ALS 6	70–75	Bulbar	12	42	3.5
ALS 7	80–85	Paraparesis	8	45	5.6

*L.O.I., Length of Illness; ALSFRS-R, ALS Functional Rating Scale-Revised; NA, Not applicable. Both the ALSFRS-R and the Progression rate were calculated at the diagnosis. The references for the Progression Rate are: slow < 0.5, intermediate 0.5–1.0, fast >1.0*.

Control subjects were individuals with subjective complaints but with no diagnosis of neurological or psychiatric disease. None of the subjects selected in this study was affected by neoplastic or autoimmune disease when the blood and CSF samples were taken.

The patients had undergone a lumbar puncture and blood sampling in the Neurology department of the Cliniques Universitaires Saint-Luc (Brussels, Belgium) as part of the routine diagnostic procedure. Patients admitted to this hospital sign an internal regulatory document, stating that left-overs from biological samples used for routine diagnostic procedures can be used for retrospective academic studies, without an additional informed consent (ethics committee approval 2007/10SEP/233). Haemorrhagic CSF samples were excluded. CSF sample collection and storage were carried out in accordance with the consensus protocol proposed by Teunissen et al. (17). For serum studies, blood samples were collected in S-Monovette® 7.5 ml Serum Z tube (ref. 01-1601, Sarstedt). Both blood and CSF were centrifuged at 3600 rpm for 6 min. Serum and cell-free CSF were respectively aliquoted and stored at −80°C for later analysis.

### GDNF, sAPP, and Aβ measurements

Secreted GDNF levels were quantified in serum and CSF from the study group by ELISA following the manufacturer's instructions (Promega). sAPPα and β and Aβ38, Aβ40, and Aβ42 peptides were quantified using a sAPPα/sAPPβ multiplex and an Aβ multiplex electro-chemiluminescence immunoassay ECLIA (Meso Scale Discovery), respectively. sAPPα and β and Aβ were quantified according to the manufacturer's instructions.

### Statistical analysis

The number of samples in each experimental condition is indicated in the figure legends. Data were analyzed using GraphPad Prism software (GraphPad Software, La Jolla, CA, USA) by unpaired Student's *t*-test (2 experimental conditions) or by ANOVA followed by Bonferroni's multiple comparison tests (>2 experimental conditions).

## Results

**Figure 1:**
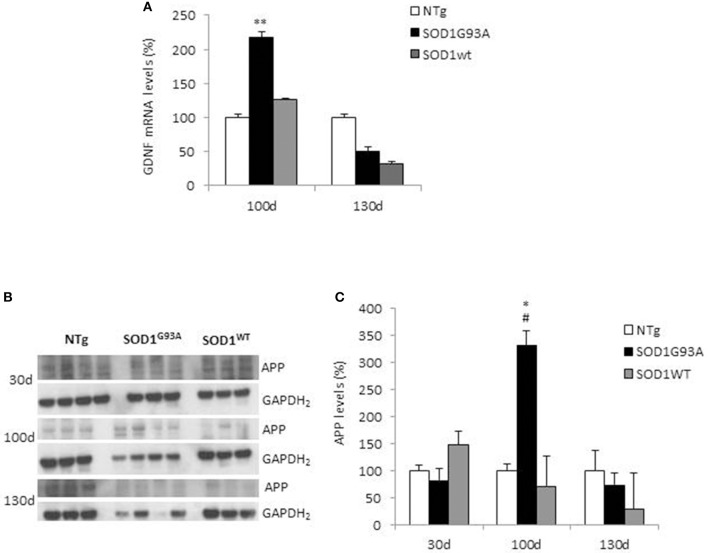
GDNF and APP levels in non-transgenic and SOD1 transgenic mice models. **(A)** GDNF mRNA levels were analyzed in hindlimbs muscles from non-transgenic (NTg), SOD1^WT^ and SOD1^G93A^ mice at ~100 (onset of motor deficits) and ~130 days of age (symptomatic stage). Values (mean ± SEM) are expressed as percentage of age-matched NTg mice. ^**^P < 0.01, ANOVA followed by Bonferroni *post-hoc* test, *n* = 3–4 mice per genotype and age. **(B)** APP levels were analyzed by Western blotting in the same muscles' lysates at 100 and 130 days, plus at 30 days (asymptomatic stage) in NTg, SOD1^WT^ and SOD1^G93A^ mice; GAPDH_2_ was used as a loading control probe. Quantifications are shown in **(C)**. Values (mean ± SEM) are expressed as percentage of expression level of age-matched NTg mice. ^*^P < 0.05 and ^#^*P* < 0.05 vs. 100-day-old genotype-matched mice NTg and SOD1^WT^; ANOVA followed by Bonferroni *post-hoc* test, *n* = 3–4 mice per genotype and age.

We analyzed GDNF mRNA levels in hindlimbs muscles from non-transgenic (NTg) or transgenic SOD1^WT^ and SOD1^G93A^ mice at 30 (asymptomatic stage), ~100 (onset of motor deficits) and ~130 days of age (symptomatic stage) (Figure 1). GDNF mRNA levels remained below the detection limit at the asymptomatic stage in all the mouse genotypes (data not shown). A significant increase in GDNF mRNA levels in SOD1^G93A^ mice vs. NTg and SOD1^WT^ mice was observed only at the onset of motor deficits at 100 days (Figure 1A), and diminished thereafter.

In the same tissues, we measured by Western blotting APP levels, which showed a significant increase in its expression in SOD1^G93A^ mice at the onset of motor deficits. More specifically, we found a 3.5-fold increase in APP levels in the hindlimb muscles at 100 days of age in SOD1^G93A^ mice compared to both NTg and SOD1^WT^ mice (Figures 1B,C). To note, the maturation profile of APP was also sharply pronounced at this stage (APP appearing as a doublet). This increase in APP expression levels was not detectable in animals aged 30 days or 130 days. By contrast, in NTg or SOD1^WT^ APP levels remained low and constant throughout the lifespan (Figures 1B,C).

Since ALS transgenic mouse models mimic the clinical situation observed in patients, we analyzed both GDNF and soluble APP fragments in biological fluids from controls subjects and ALS patients. Group characteristics of study population are shown in Table 1. The mean age of the control group was 58.6 ± 9.9 years (range 43–75 years) and that of ALS group was 64.1 ± 13.5 years (range 38–81 years). There was no significant statistical difference between the groups (*p* = 0.398). The mean duration of disease in ALS patients was 13.4 ± 15.7 months; the mean ALSFRS-R and the Progression rate calculated at the diagnosis were 41.7 ± 3 and 9.9 ± 15.1 respectively, indicating that ALS patients were all fast progressors, with one intermediate and no slow progressors.

**Figure 2:**
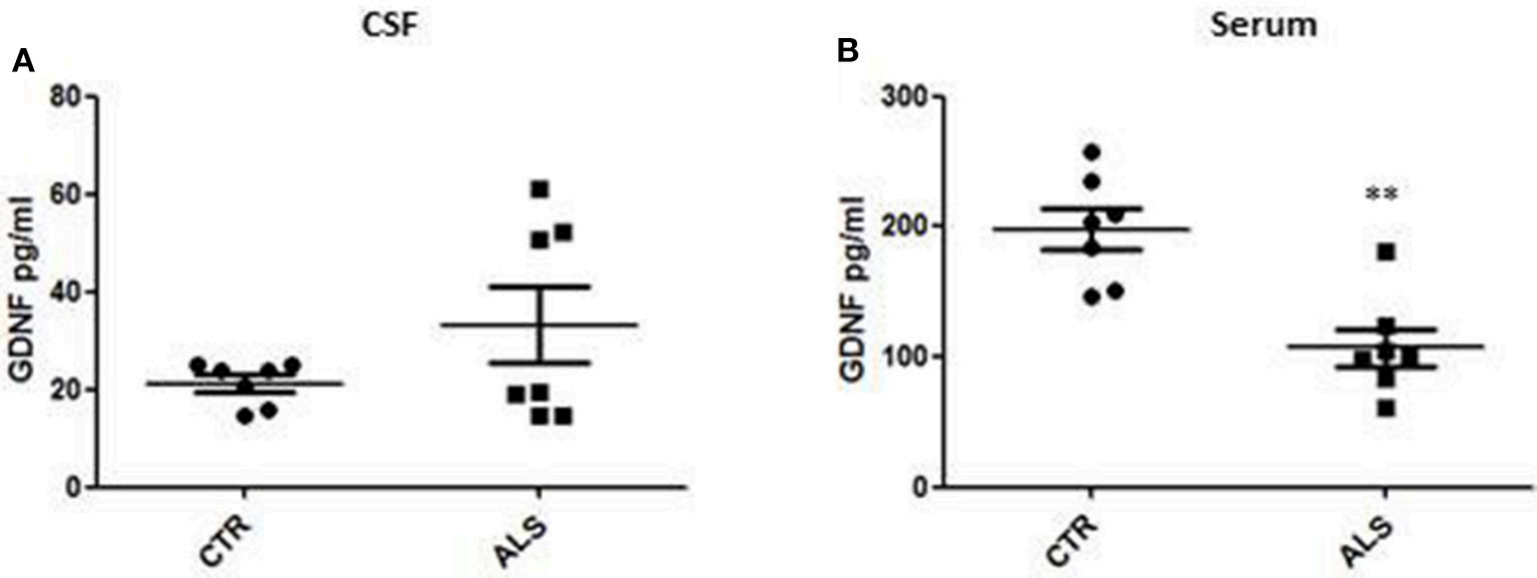
GDNF levels in biological fluids from CTR subjects and ALS patients. GDNF levels were quantified by ELISA in the CSF **(A)** and serum **(B)** of controls without neurological disease and ALS patients. Values are given in picograms per milliliter (pg/ml). Each dot corresponds to one subject studied; the horizontal bar indicates the mean in each group. ^**^P < 0. 001, Student's *t*-test (*n* = 7/group).

**Figure 3:**
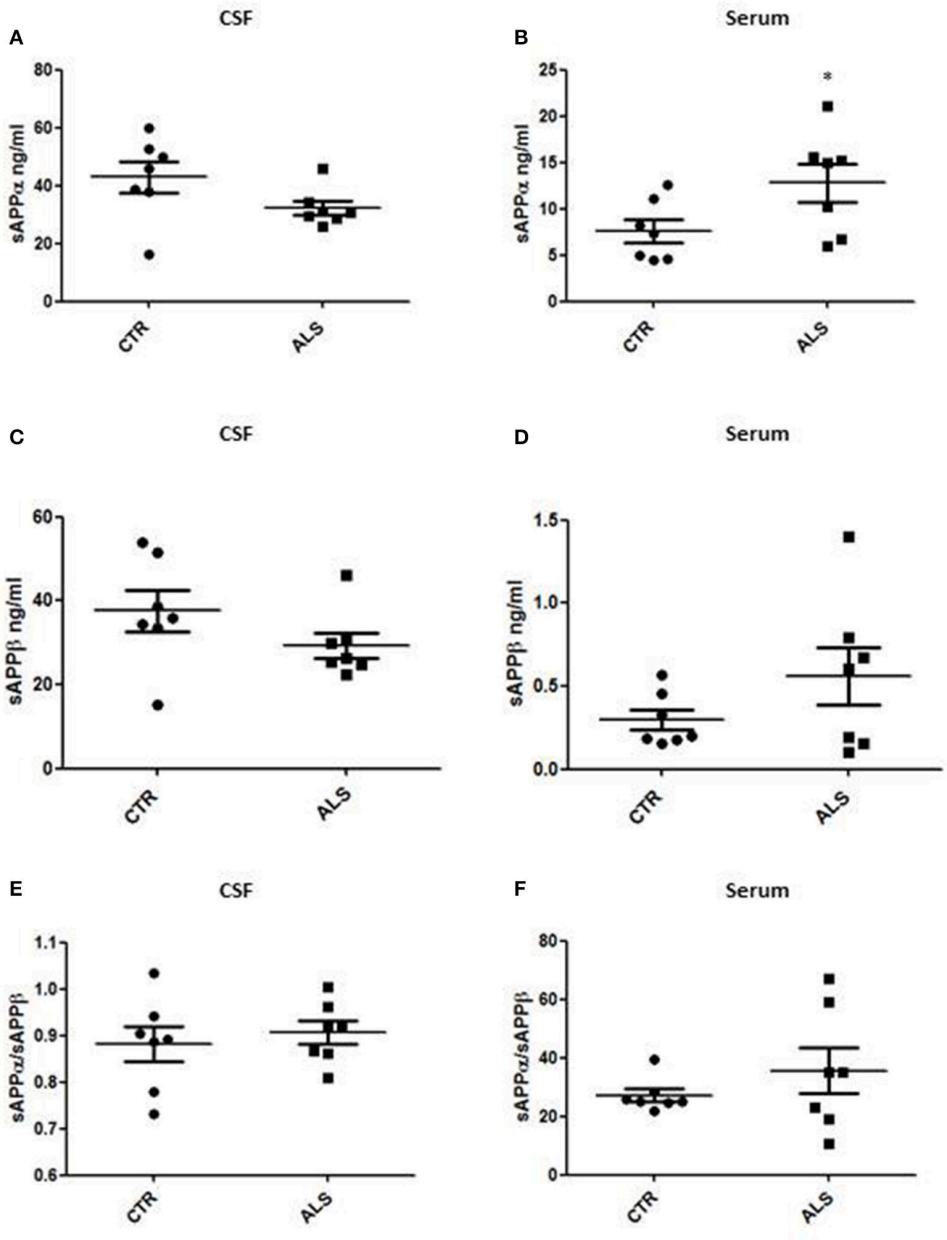
CSF and serum sAPPα, β and their ratio in CTR and ALS patients. sAPP α levels **(A,B)** and sAPP β **(C,D)** were quantified by ECLIA in the CSF and serum of controls without neurological disease and ALS patients. sAPP α / sAPP β ratio have been showed for both CSF **(E)** and Serum **(F)**. Values are given in nanograms per milliliter (ng/ml). Each dot corresponds to one subject studied; the horizontal bar indicates the mean in each group. ^*^P < 0.05, Student's *t*-test (*n* = 7/group).

**Figure 4:**
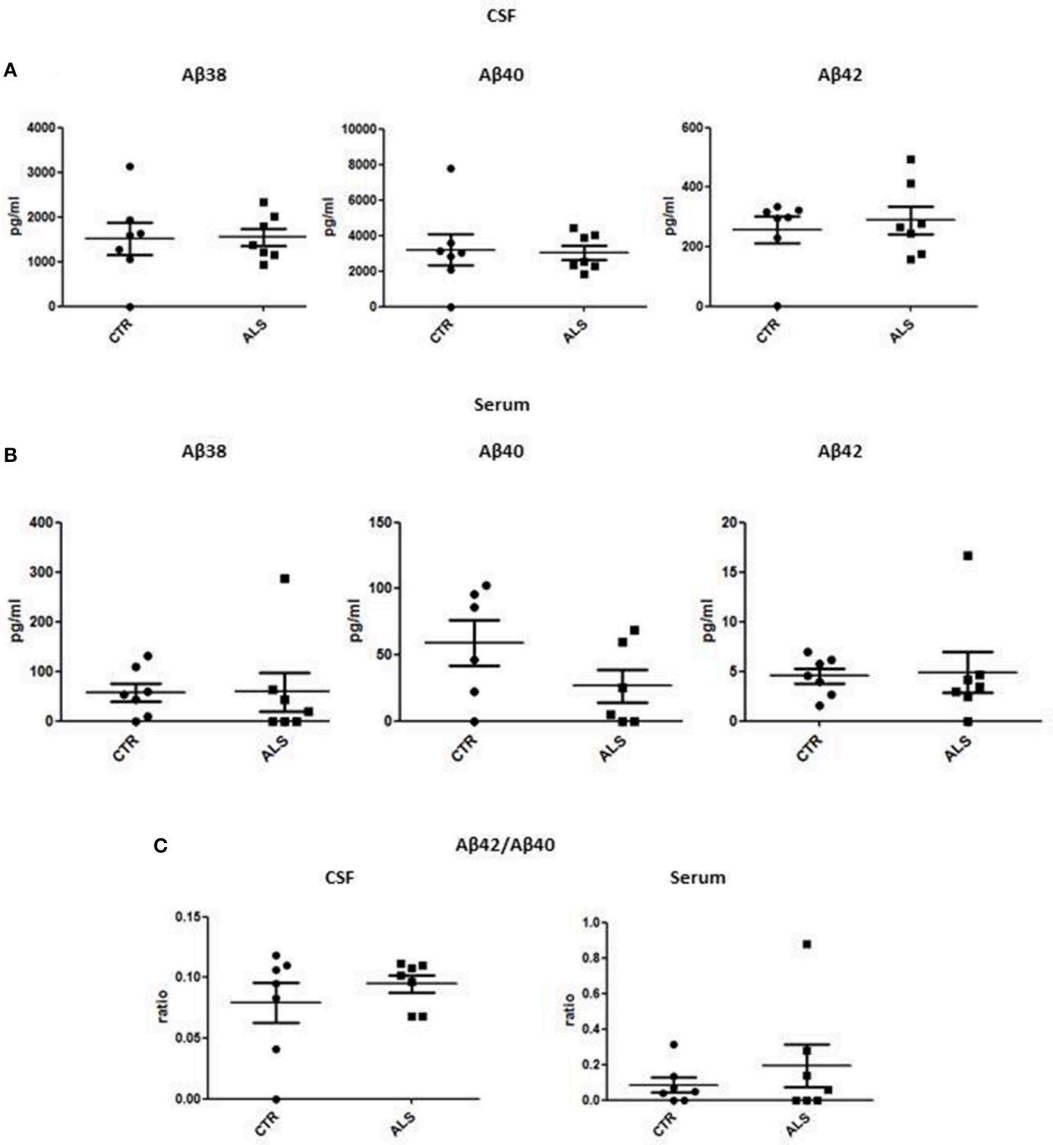
CSF and serum Aβ38, Aβ40, and Aβ42 and Aβ42/Aβ40 ratio in CTR and ALS patients. Soluble monomeric Aβ38, Aβ40, and Aβ42 were quantified by ECLIA in the CSF **(A)** and serum **(B)** of controls without neurological disease and ALS patients. **(C)** Aβ42/Aβ40 ratio have been showed for both CSF and Serum. Values are given in nanograms per milliliter (ng/ml). Each dot corresponds to one subject studied (*n* = 7/group); the horizontal bar indicates the mean in each group.

We measured GDNF concentration in CSF and serum from healthy subjects and ALS patients to follow their expression in biological fluids reflecting the biochemical changes ongoing in the brain and in the peripheral tissues, respectively. The median of GDNF levels in CSF was higher in ALS patients (33.1 ± 20.5 pg/ml) compared to CTR (21.2 ± 4.3 pg/ml), but the statistical significance was reached only in serum, where ALS patients showed clearly lower levels of GDNF when compared to CTR (107.3 ± 37.7 pg/ml vs. 198.1 ± 41 pg/ml, respectively). These results (Figures 2A,B) were consistent with the decrease in GDNF expression oberserved in SOD1^G93A^ mouse muscles at the advanced stage (Figure 1A). APP holoprotein cannot be tracked in extracellular media. In order to analyse a potential correlation with the amount of soluble APP levels in subjects' biological fluids and GDNF levels, we measured by ECLIA the soluble non-amyloidogenic fragments of APP: sAPPα and β, both in CSF and serum (Figures 3A–D) and their ratio (Figures 3E,F) that provides additional information about the equilibrium between non-amyloidogenic and amyloidogenic APP processing. Interestingly, the levels of sAPPα were increased only in serum of ALS patients compared to controls. No differences were observed in CSF. To note, neither sAPPβ levels nor sAPPα/β ratio was affected in serum or CSF of patients with ALS. Concerning the levels of Aβ38, Aβ40, and Aβ42, in both CTR and ALS patients, Aβ peptides were detectable only in CSF (Figure 4A), while in serum Aβ levels were very low (Figure 4B). No significant differences were observed in Aβ42/Aβ40 ratio (Figure 4C).

## Discussion

The lack of clinical criteria to accurately identify the “at-risk group” for ALS development together with the unknown etiology of the disease are fueling the interest in biomarkers aimed at completing clinical approaches for the diagnosis and suitable as new therapeutic targets. In this study, we explored specifically if the loss of trophic support in neurodegenerative diseases could provide *bona fide* biomarkers for ALS. We measured GDNF, APP as well as soluble APP fragments and Aβ peptides levels, in mouse models of ALS and in human biological fluids (i.e., serum and CSF) from patients and control subjects.

Glial cell line-derived neurotrophic factor is a diffusible peptide critically involved in neuronal differentiation and survival and it has been identified in an unbiased proteomic assay as potential AD biomarker (18, 19). It has been evaluated for symptomatic treatment of Parkinson's disease (PD) (20), and shown to be able to reverse some aspects of aging in monkeys (21). Importantly, GDNF is particularly involved in the pathophysiology of various neurological and neuromuscular diseases, with a great interest in the peripheral nervous system (PNS). GDNF has been reported to preserve motor neurons from dying by using neural progenitor cells delivery (22) or muscle-derived GDNF (23), suggesting that therapies targeting GDNF could be efficient to cope with ALS. Here, we have reported a clear alteration of GDNF levels both in muscles from ALS mouse models and serum from ALS patients. We found an increase in GDNF levels in muscle from mice expressing the mutant isoform of SOD1^G93A^ at the onset of symptoms (100 days). Other groups observed an increased expression of GDNF mRNA in skeletal muscle from patients (24–26). The increment in GDNF mRNA levels in SOD1G93A mice is concomitant to the onset of ALS symptoms (denervation) but it is temporary. As it has been demonstrated in patients; GDNF levels increase contemporary to denervation aiming to promote potential reinnervation, but the reaction may be transient. We believe that the decrease in GDNF mRNA levels observed at day 130 might be due to the reduction in the total number of muscle fibers, the low number of fibers in acute stages of neurogenic atrophy and replacement of muscle fibers by connective tissue occurring in later stages of the disease. The fact that a similar reduction in GDNF mRNA levels is detected in SOD1wt mice can be explained by the presence of motor neuron pathology and skeletal muscle atrophy also in these animals (27, 28).

All together, these findings suggest that GDNF is importantly synthetized by muscles to sustain the increased demand for trophic factors by motor neurons, which are prone to degenerate in ALS. Interestingly, we observed also a significant increment in APP levels in mice expressing the mutant isoform of SOD1^G93A^ at the stage of disease onset (100 days). This is in line with previously published work (13, 14). We recently found that APP controls GDNF transcription in muscles, supporting the neuromuscular phenotype observed in APP knock-out (KO) mice (12). This result suggests that changes in APP expression observed herein and in muscles of patients with ALS (14), could directly affect muscular GDNF expression and release. Thus, APP could not only be a biomarker of ALS progression, but it could be involved in the pathways controlling trophic supply that are impaired in the ALS pathology.

In human samples, we observed significant and clear-cut results in serum while we measured only tendencies of impairment for both GDNF and APP metabolites in CSF. More specifically, we recorded a trend of GDNF levels to increase in the CSF of only 3 over 7 ALS patients, while a uniform and significant decrease in serum from ALS patients was detected. Our results indicate that peripheral GDNF (serum) is decreased as consequence to the fact that the regulatory system of GDNF production, likely involving APP, is no more functional and therefore reflected by a decrease of GDNF in serum. This is a peripheral process since central GDNF (CSF) is not significantly affected in the pathology. In parallel, we studied APP metabolite levels in the patient samples and we observed a trend to decrease of sAPPα levels in the CSF of ALS patients and a significant increase in sAPPα levels in serum from patients. Interestingly, sAPPα has been suggested to have potent neuroprotective capacities (29). Alterations in sAPPα levels in peripheral fluids from patients could indicate that peripheral mechanisms involving APP-dependent GDNF regulation may be implicated in the disease. Same observations, though not statistically significant, hold true for sAPPβ while no differences have been detected neither in sAPPα/sAPPβ ratio nor in Aβ levels.

Aβ levels are one of the most extensively evaluated markers of sporadic AD, since GDNF was identified as potential AD biomarker (18, 19), could we suggest it as a general biomarker of neurodegenerative diseases? The question is open. What is clear is that in AD Aβ levels are importantly high, while in ALS there are no alterations compared to age-matched control subjects at any stage of the disease. This suggests that very likely, APP and GDNF are linked in different neurodegenerative diseases but the mechanistic aspects are peculiar to the specific disease and still need to be decipher.

It is at this stage difficult to make clear-cut correlations between GDNF and APP soluble fragments in human samples because of the reduced number of ALS patients enrolled in the study, the variability existing between them and especially because of the peculiarity of disease history. Large scale studies on bigger cohorts would be useful to elaborate on the observations we made here. Clearly, both GDNF and soluble sAPPα levels are altered in fluids from patients with intermediate and fast progression of the disease, indicating that GDNF and soluble APP are biomarkers of ALS pathophysiology. The unequivocal observations in mouse muscles and serum from patients strongly suggest that changes in APP and GDNF levels result from peripheral processes. Assessing biomarkers in blood has the great advantage of minimal invasiveness when compared to measurement in CSF samples.

Changes in GDNF and sAPPα in serum go in opposite directions leaving open the following question: is APP-dependant GDNF expression involved in ALS progression, or are they independent biomarkers? The correlation between APP-related and GDNF changes, and their contribution to ALS pathways need to be fully elucidated, in order to consider them as possible targets for therapeutic approaches.

In conclusion, we suggest that the combined analysis of GDNF and sAPPα that we propose as possible predictive peripheral biomarkers for ALS, could help for the comprehension of the etiopathogenesis and the improved precision of the diagnosis of ALS.

## Author contributions

SS and PK-C designed the research study. SS conducted experiments, LB, BT, and AD offered technical help. VvP enrolled subjects and conducted the clinical diagnosis. SS wrote the paper with fundamental input of J-NO, DR, and P-KC. All the authors analyzed data, read and approved the final manuscript.

### Conflict of interest statement

The authors declare that the research was conducted in the absence of any commercial or financial relationships that could be construed as a potential conflict of interest.
